# Practice Patterns and Trends in Temperature Control After Cardiac Arrest: A Multi-Specialty Survey

**DOI:** 10.3390/jcm14238592

**Published:** 2025-12-04

**Authors:** Casey T. Carr, Melody B. Eckert, Nilan Bhakta, Faheem W. Guirgis, Charlotte Hopson, Carolina B. Maciel, Torben K. Becker

**Affiliations:** 1 Nazih Zuhdi Transplant Institute, Specialty Critical Care and Acute Circulatory Support Service, INTERIS Baptist Medical Center, Oklahoma City, OK 73112, USA; 2 College of Medicine, State University of New York Downstate Health Sciences University, New York, NY 11203, USA; 3 College of Medicine, University of Texas Health Science Center at Houston, Houston, TX 77030, USA; 4 Department of Emergency Medicine, College of Medicine, University of Florida, Gainesville, FL 32610, USA; 5 Departments of Neurology and Neurosurgery, College of Medicine, University of Florida, Gainesville, FL 32610, USA; 6 Division of Critical Care Medicine, Department of Emergency Medicine, College of Medicine, University of Florida, Gainesville, FL 32610, USA

**Keywords:** targeted temperature management, temperature control, therapeutic hypothermia, cardiac arrest, post cardiac arrest syndrome

## Abstract

**Background/Objectives:** Temperature control after cardiac arrest remains a recommended component of post-cardiac arrest care, yet substantial practice variability persists. Conflicting evidence regarding optimal temperature targets and mixed interpretations of recent trials, such as TTM2, may contribute to inconsistent bedside implementation. Understanding physician knowledge, attitudes, and practice patterns is essential for aligning post-cardiac arrest management with evolving evidence. This study aimed to characterize international physician perceptions of temperature control, patterns of use, understanding of neurologic injury, and the influence of emerging literature. **Methods:** A 39-item web-based survey was developed through iterative expert review and pilot testing and disseminated to members of critical care, neurology, and emergency medicine societies between September 2021 and January 2022. The instrument assessed demographics, temperature control practices, interpretation of new literature, and post-cardiac arrest management. Responses were analyzed using descriptive statistics in R Studio, with proportions reported for categorical variables and mode responses for ranked questions. **Results:** Among 501 respondents, 471 (94%) completed the survey. Most were attending-level physicians (73%), primarily practicing intensive care medicine (75%), and based in academic centers (60%). Targeted temperature management (TTM) was commonly initiated by the admitting intensivist (66%), most often because guidelines recommended it (67%). The most influential factors driving initiation were institutional protocols (21%), perceived neurologic prognosis (17%), and arrest etiology (14%). The most frequently selected temperature target was 36 °C (44%). Awareness of the TTM2 trial was high (70%), though only 31% reported altering their practice in response. Neurologists were more likely to individualize temperature targets and select lower temperatures, while physicians caring for higher cardiac arrest volumes also favored lower targets. Community clinicians more commonly selected lower temperature targets compared with those in academic settings. **Conclusions:** Substantial heterogeneity exists in the practice and rationale for temperature control after cardiac arrest. Physician specialty, cardiac arrest volume, and local practice environment influence the temperature target selection and attitudes toward emerging evidence. Despite awareness of new data, institutional protocols remain the dominant factor guiding implementation. Standardized, evidence-based institutional pathways may help reduce practice variability and promote consistent post-cardiac arrest care.

## 1. Introduction

In the management of patients who have survived cardiac arrest, temperature control—with a broad range of temperature targets—remains a key guideline-recommended tool for improving outcomes during post-cardiac arrest care of successfully resuscitated patients [1,2]. While the early literature showed a neurologic benefit [3,4], recent studies challenge the practice of the contemporary use of temperature control [5,6,7], suggesting that rather than induced hypothermia, avoidance of fever is key [5]. There is significant variability in the implementation of temperature control, despite recommendations by international societies [1,2,8]. In an institutional survey, we found a significant contribution of physician understanding and perceptions as barriers to temperature control implementation, suggesting that physician attitudes as the primary reason behind practice variability in temperature control [9]. Additionally, as data continue to emerge regarding temperature control and patient-oriented outcomes, how this science is interpreted and used at the bedside is unclear and remains an unanswered question [10].

While it is clear that additional nuanced approaches to therapeutic targets must be investigated and discussed, the inconsistent practice of temperature control remains a perplexing challenge. It is possible that the relevant literature is not reaching front-line clinicians or that the trajectory of neurologic injury in cardiac arrest is misunderstood. The conflicting evidence regarding temperature control likely contributes to the variability in its use. In addition, there could be additional factors in the miasma of cardiac arrest that clinicians encounter that discourage the use of temperature control—factors such as the perception of delay in prognostication, resource limitations, the presence of exclusion criteria, and the lack of institutional protocols.

Understanding the lack of guideline adherence is critical in promoting the evidence-based use of temperature control. Additionally, investigating physicians’ understanding of the neurologic sequelae of cardiac arrest, which is fraught with complexities and challenges, has not previously been performed and may help explain additional issues in post-cardiac arrest care, such as early withdrawal of life-sustaining therapies [11]. The motivation and need to understand the etiology of the variability in post-cardiac arrest management is based upon the scientific goal to align bedside clinical practice with societal guidelines and the emerging medical literature. Understanding the gap between practice and the literature allows for the opportunity to intervene on this discordance—and perhaps seek to understand what bedside clinicians know that professional societies and researchers do not.

Through an international survey, we aimed to investigate physicians’ attitudes and perceptions of temperature control, how temperature control is utilized at the bedside, physicians’ understanding of post-cardiac arrest neurologic injury, and how new evidence influences practicing clinicians.

## 2. Methods

### 2.1. Survey Instrument Development

The survey instrument was developed in multiple phases (Phase 0 and Phase 1), starting with a locally derived survey instrument that was subsequently published and then further refined by an expert focus group.

The previously published original survey instrument [9] (Phase 0) was developed based on expert opinion, local practice, and committee discussion using Qualtrics™ (Seattle, WA, USA). Item and question generation revolved around several major arms of the survey. The first arm of the survey comprised 8 questions pertaining to respondent demographics and background. The second arm comprised 19 questions specific to TTM practice, including the influence of newly published TTM literature. The third arm of the instrument contained 11 questions ascertaining post-cardiac arrest practices, and the final arm of the instrument included 5 questions specific to post-cardiac arrest coronary reperfusion strategies and practices.

This instrument was first utilized in a local survey regarding institutional practice patterns and was published separately [9]. Following the disbursal and publishing of this instrument, additional refinement and iteration development occurred via expert focus group feedback (Phase 1). This feedback went to further refine question diction, reduce redundancy, and reduce item choice. This instrument was administered to all institutional intensivists for formal pilot testing and initial statistical analysis (Phase 2). The response rate in this pilot test was approximately 23% (16/69). The results of Phase 2 were analyzed for internal consistency (Supplementary Materials Table S1).

The CHERRIES validated checklist for reporting e-surveys was utilized to ensure the standardization of survey data reporting [12].

The final survey instrument was a 39-question web-based survey via Qualtrics™ (Seattle, WA, USA). As this was an exploratory study, no a priori power calculation for study size was performed.

### 2.2. Instrument Disbursal

The developed survey instrument was distributed to multiple academic societies, which included key stakeholders and physicians who regularly performed post-cardiac arrest care and temperature control—Society of Critical Care Medicine, European Society of Intensive Care Medicine, American Academy of Neurology, and Neurocritical Society. Disbursal methods varied and included emails to member listservs and message board systems. Given this disbursal strategy, the number of total recipients could not be calculated, precluding response rate calculation. Respondents were allowed to choose more than one specialty, and when this occurred, respondents were sorted via their non-ICM-selected specialty. The initial survey disbursal occurred in September 2021 and closed in January 2022.

### 2.3. Statistical Analysis

The survey data was analyzed in R Studio™ (Version 12; Vienna, Austria). We calculated counts and proportions for the total cohort for multiple-choice questions and reported the mode response for questions where the responder was asked to choose the “top” or “most common” relevant response. In one question, the responders were asked to rank possible factors leading to the initiation of temperature control in their practice—we determined and reported the three responses that were most likely to be ranked first.

Stacked proportional bar charts displaying the proportion of responses for the target temperature used in temperature control by number of cardiac patients seen and by practice setting were created using the ggplot2 package (Version 3.4.4; Vienna, Austria).

## 3. Results

There were 501 total respondents, with 471 (94%) respondents who finished the survey; an additional 154 (30.1%) started the survey but did not achieve completion in totality. Three respondents did not consent to the survey, and 86 (17.1%) answered one question or fewer, so they were not included in the results.

### 3.1. Demographics of Respondents

The majority of intensivists (Table 1) regularly cared for patients after cardiac arrest, most commonly greater than 50 patients per year (31%), and cared for post-cardiac arrest patients every month (43%) and as frequently as every week (41.2%). Most respondents were attending/consultant level (73%), primarily intensive care medicine-specialized (39.2%), based in the United States (86%), and practiced in primarily academic settings (60%).

### 3.2. Targeted Temperature Management Initiation (Table 2)

The admitting intensivist was responsible for decision-making regarding temperature control initiation in the majority of cases (66%). The leading reason for starting temperature control was that it was “recommended by national/international guidelines” (67%), with the most influential factors being “institutional protocol” (21%), “perceived neurologic prognosis” (17%), and “etiology of cardiac arrest” (14%). The most commonly targeted temperature was 36 °C (44%).

**Table 2:** Factors affecting decision-making in temperature control.

Temperature Decision Factors		
Survey Question	Survey Answer	
Who is responsible for initiating TTM?	Accepting Intensivist	331 (66%)
	Consulting Intensivist	44 (9%)
	Emergency Physician	52 (10%)
	Neurological Consultant	21 (4%)
	Other	14 (3%)
	Unknown/No response	39 (8%)
Have you ever started TTM/TH in a post-cardiac arrest patient?	Yes	441 (88%)
	No	26 (5%)
	No response	34 (7%)
Have you ever thought about starting TTM/TH but did not?	Yes	272 (54%)
	No	136 (27%)
	Unknown/No response	93 (19%)
Leading choice for starting TTM:	It is recommended by national/international guidelines (336, 67%)	
Three most influential factors (ranked as top factor) leading to initiation of TTM/TH in post-cardiac arrest patients at your primary hospital:	1. Institutional protocol (106, 21%) 2. Perceived neurological prognosis (84, 17%) 3. Etiology of cardiac arrest (69, 14%)	

### 3.3. Response to the Emerging Literature (Table 3)

Of the respondents, 25% reported recent changes in temperature control practices in the past 5 years, with the most common change being a change in targeted temperature. The majority of the respondents were aware of the TTM2 Trial (70%)—31% of respondents reported this trial changed their practice, while 39% reported that it would not change their practice.

**Table 3:** Changes in practice and attitudes regarding TTM2 trial.

Changes in Temperature Management		
Survey Question	Survey Answer	
Have there been changes in how TTM/TH is performed at your institution in the past 5 years (prior to the TTM2 trial)?	Yes	154 (31%)
	No	127 (25%)
	Unknown	220 (44%)
What has been the most common change?	Target normothermia (<37.5) rather than hypothermia (<36) (115, 23%)	
Are you aware of the publication/results of TTM2 trial?	Yes	353 (70%)
	No	72 (14%)
	No response	76 (15%)
Have the results of the TTM2 trial changed your practice of TTM/TH?	Yes	156 (31%)
	No	194 (39%)
	No response	151 (30%)
What is the most common reason that has limited the incorporation of the TTM2 trial results into your practice?	Knowledge of conflicting/divergent data (82, 16%)	

### 3.4. Subgroup Analysis Across Specialty, Cardiac Arrest Volume, and Academic Setting (Figure 1 and Figure 2)

The most common specialty selected was Intensive Care Medicine (75%). Anesthesiology and Surgery specialists selected 33 °C as a target temperature rarely (28% and 30%, respectively), while respondents who specialized in Neurology selected 33 °C most commonly (45%). Intensive Care Medicine (ICM) specialists selected 33 °C at a 37% rate.

Neurology was the most likely to individualize target temperatures (70%), while Anesthesiology was the least likely (25%) to do so.

All specialties initiated temperature control for the most common reason, which was that “It is recommended by national/international guidelines.” However, neurology had an equally frequent rate of “We wanted the best neurological recovery.” The most influential factor for all specialties for initiation of TTM was “Institutional protocol.”

**Figure 1:**
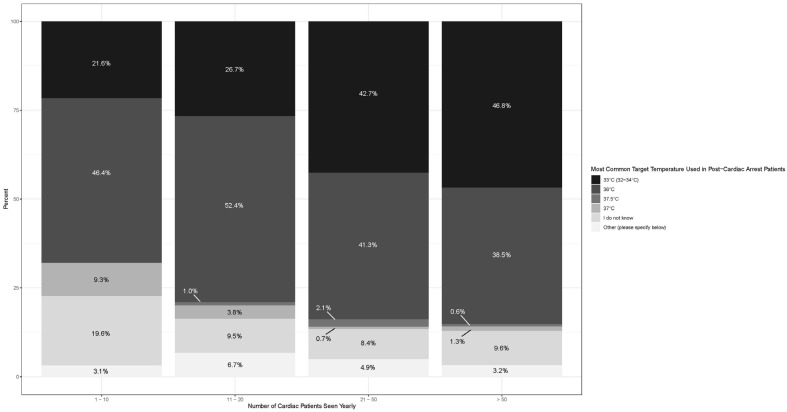
Temperature targets in relation to cardiac arrest case volumes.

**Figure 2:**
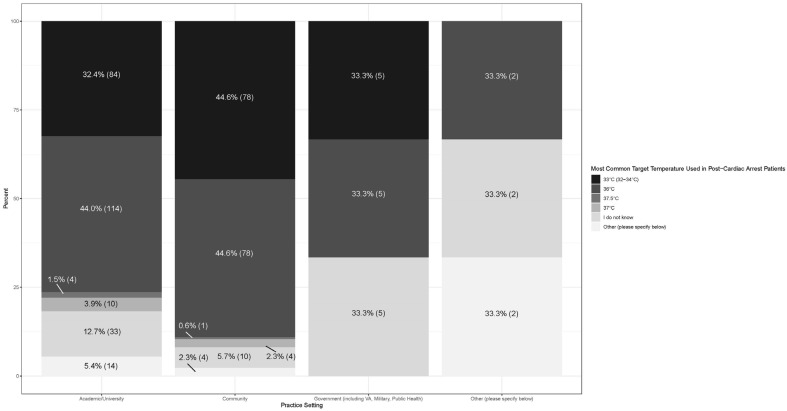
Temperature targets in relation to practice setting.

Higher cardiac arrest volumes (>50 patients per year) were associated with lower temperature targets (Figure 1), and as cardiac volumes increased (from >10 to >20 to >50), the goal temperature decreased. Similarly, community practice settings had a higher rate of lower targeted temperatures than academic practice settings (Figure 2).

## 4. Discussion

Our results show that there continues to be great variation in the use of temperature control after cardiac arrest—there is no cohesive target temperature, no consensus on the interpretation of TTM2, and a wide variety of indications for TTM. Various individual factors are associated with trends toward temperature control practice—experience, higher volumes of cardiac arrest cases, and primary specialty.

Temperature control in the post-cardiac arrest setting has continued to evolve over the course of the past several years, with a notable dynamic landscape and a shift in the range of targeted temperatures. This evolution accompanied ongoing changes to the overall management in the post-arrest setting [13]. These changes in the scientific landscape have led to practice variation, various levels of guideline adherence, and potential differences in patient outcomes.

To our knowledge, this is the first study surveying the international practice of temperature control. Khera et al. [14] investigated the use of targeted temperature management on a retrospective and system-wide basis, noting wide variation and discrepancies between clinical sites, including significant variation from recommended practices. These data demonstrated system-level differences across a consortium of many hospitals, comparing patients who were candidates for temperature control. Our data supports the finding of variable temperature control practices, but on a physician-level basis. We have shown that physicians often rely on institutional protocols and guidelines and often prioritize temperature control when there is a perception that temperature control improves neurologic outcomes.

Our data demonstrates that while guidelines and recently published literature frequently influence practitioner behavior and attitudes, what is often most relied upon are institutional-level protocols. This lends itself to the conclusion that establishing these protocols is incredibly important for institutions that have none, similar to other forms of bundled care [15]. While our data was not meant to study patient outcomes, there is clear data that shows protocol implementation improves logistical results, and likely patient outcomes as well [16,17]. Additionally, as scientific bodies attempt to create change and minimize variation in the care of post-cardiac arrest patients, encouraging the creation of institutional protocols may be a key component. As we aim to improve outcomes for all survivors of cardiac arrest and minimize variations in management, identifying the importance of protocol implementation is critical knowledge.

In addition, Garfield et al. [18] studied the effects of the newly published temperature control literature, albeit in a single-center, retrospective “before and after” study [6]. They showed that temperature control use declined, with an increased risk of fever, during the study period of 7 years. Our data is somewhat discordant, but was conducted in a different era—that of the TTM2 trial. Only half of the respondents in our cohort were likely to change their practice after the TTM2 trial, suggesting a continuation in the perception of equipoise in the temperature control scientific landscape. This warrants further discussion and analysis, specifically regarding the interpretation of this data and resistance to the conclusion of the trial.

Interestingly, there were several associations between temperature control practice and respondent types. Respondents who identified as “Neurology” were more likely to both select a lower temperature target and to individualize temperature control therapies. Additionally, neurologists had the highest selected reasoning: “We wanted the best neurological recovery.” While it is not particularly surprising that practitioners specializing in neurology would emphasize neurological recovery, the difference in actual temperature control practices is noteworthy. Perhaps the potential downsides of TTM, such as hemodynamic compromise, metabolic shifts, and delayed awakening, are perceived as too great a cost by non-neurologist intensivists, whereas the benefits of temperature control are weighed more heavily by neurointensivists. Or perhaps non-neurology-based physicians are less focused or aware of hypoxic–ischemic encephalopathy. Additionally, neurologists are likely more aware of tools that can be used to individualize temperature control therapies or even that the practice is an option. These findings underlay the importance of multi-disciplinary care in the management of post-cardiac arrest patients—involvement of a neurologist may change individual patient recommendations and management, or at the very least, lead to discussion regarding individualizing temperature control goals.

Practitioners who managed higher volumes of cardiac arrest patients also more often initiated temperature control at a lower temperature goal. This aligns with the finding that specialized, high-volume centers are often more frequently adherent to cardiac arrest management guidelines and more frequently offer advanced therapies [19].

## 5. Limitations

There are natural limitations to our data, given the nature of survey-based science, such as the presence of unanswered questions, inaccurate responses that may reveal differences between respondent answers and actual practice, the fact that different specialists may have different roles and responsibilities based on country and setting of practice, and the number of responses varying by discipline, as control over representation from each discipline was not possible. Additionally, there was a minority of surveys that were started and not completed; however, this was a small amount (*N* = 29) and only represented 6% of the respondents, so incomplete surveys would be very unlikely to have influenced the results. We also selected our disbursement target based on governing bodies, which may have led to a selection bias in the respondents, and it also did not allow for the calculation of a response rate. This likely creates a bias towards those most interested in and experienced with post-cardiac arrest care, or those who have a specific opinion regarding temperature control. Also, there are likely specialties and physicians who do participate in post-cardiac arrest care, but would not be captured by our survey strategy, such as family medicine or hospital medicine. Despite being disbursed on an international level, the survey instrument was only available in English, and there is a preponderance of respondents from the United States and Western European countries. This potentially could bias results to post-cardiac arrest care that does not reflect both clinician practice and patient populations in non-Western settings, as well as in lower resource settings.

## 6. Conclusions

Physicians and other practitioners who manage patients after cardiac arrest have a significant heterogeneity in the reasoning, practice, and implementation of temperature control. Certain respondent characteristics, such as patient volumes and primary specialty of practice, influence temperature control decisions. Physicians rely heavily on institutional protocols when initiating temperature control after cardiac arrest.

## Figures and Tables

**Table 1:** Demographic characteristics of respondents.

Respondent Demographics		
Survey Question	Survey Answer	
Approximately how many cardiac arrest patients do you see at your primary hospital of practice each year?	1–10	97 (20%)
	11–20	105 (21%)
	21–50	143 (29%)
	>50	156 (31%)
What is your role?	Attending/Consultant	366 (73%)
	Fellow/Resident/Registrar	41 (8%)
	Other	94 (19%)
Specialty area of practice (multiple possible):	Anesthesiology	60 (12%)
	Cardiology	17 (3%)
	Emergency Medicine	51 (10%)
	Intensive Care Medicine	375 (75%)
	Internal Medicine	34 (7%)
	Neurology	53 (11%)
	Pediatrics	40 (8%)
	Surgery	33 (7%)
	Other	40 (8%)
Year of graduation from professional medical training (after completion of residency or fellowship):	Before 1980	13 (3%)
	1980–1989	37 (7%)
	1990–1999	73 (15%)
	2000–2009	106 (21%)
	2010–2019	209 (42%)
	2020 or later	63 (13%)
Type of practice setting (multiple possible):	Academic/University	303 (60%)
	Community	213 (43%)
	Government	25 (5%)
	Other	6 (1%)
Approximate number of beds in primary hospital of practice:	<200	43 (9%)
	200–499	196 (39%)
	500–1000	202 (40%)
	>1000	55 (11%)
	Unknown	5 (1%)

## Data Availability

The data presented in this study are available on request from the corresponding author due to the presence of protected health information and the need to protect respondent privacy.

## Supplementary Materials

The following supporting information can be downloaded at: https://www.mdpi.com/article/10.3390/jcm14238592/s1, Table S1: Internal Survey.
