# Supplementary material for: Practice Patterns and Trends in Temperature Control After Cardiac Arrest: A Multi-Specialty Survey
Source: J Clin Med. 2025 Dec 4;14(23):8592. doi: 10.3390/jcm14238592 (PMC12693480; doi:10.3390/jcm14238592)
Supplement: Supplementary file 1 [file jcm-14-08592-s001.zip › jcm-3995452-supplementary.pdf]

Waiver

Title of Study: Survey of Practices and Physician Attitudes on Post-Cardiac Arrest Care (IRB202002210)

Principal Investigators:
Torben K. Becker MD PhD
Casey T. Carr MD
Department of Emergency Medicine
University of Florida

1. **Purpose of the Study:** The purpose of this study is to analyze current practices and attitudes regarding post-cardiac arrest care, specifically targeted temperature management and coronary reperfusion strategies.

2. **Procedures to be Followed:** You will be asked to answer several questions on a survey.

3. **Duration:** It will take 5-7 minutes to complete the survey.

4. **Research Benefits:** There is no direct benefit to you for participating in this study.

5. **Research Risks:** This is an anonymous survey and there are no anticipated risks.

6. **Statement of Confidentiality:** Your participation in this study is confidential. The survey does not ask for any information that would identify to whom the responses belong. In the event of any publication or presentation resulting from the research, no personally identifiable information will be shared.

7. **Data Security:** The data of this project will be stored in electronic form using the Qualtrics platform which uses advanced encryption software.

8. **Right to Ask Questions:** Please contact Dr. Torben Becker at t.becker@ufl.edu with

questions or concerns.

9. **Privacy Authorization:** UF will collect the data asked in this survey only for this study and will not share them with researchers unaffiliated with this project.

10. **Voluntary Participation:** Your decision to participate in this study is voluntary. You can stop at any time. You do not have to answer any questions you do not want to answer. If you have already completed this survey through another professional society, please do not participate again. If you have any questions about your rights as a research subject, you can phone the Institutional Review Board at 352-273-9600.

Do you consent to participate in this research study?

- Yes
- No

Demographics

Approximately how many cardiac arrest patients do you see at your primary hospital of practice each year?

- 0
- 1 - 10
- 11 - 20
- 21 - 50
- > 50

How often on average do you care for patients who have been resuscitated after cardiac arrest?

- Every week
- Every month
- A few times a year
- Rarely
